# Stabilization of U(V) and U(VI) in Goethite Formed by Recrystallization of Fe-Oxyhydroxysulfates

**DOI:** 10.1021/acs.est.6c02403

**Published:** 2026-05-19

**Authors:** Liubov Kononova, Mats Åström, Elena F. Bazarkina, Damien Prieur, Kristina O. Kvashnina, Tao Luo, Jean-François Boily, Henrik Drake, Viktor Sjöberg, Changxun Yu

**Affiliations:** † Centre for the Environment (CENWIN), 4180 Linnaeus University, 39231 Kalmar, Sweden; ‡ The Rossendorf Beamline (BM20), The European Synchrotron, 38043 Grenoble, France; § Institute of Resource Ecology, 28414 Helmholtz-Zentrum Dresden-Rossendorf, 01328 Dresden, Germany; ∥ Department of Chemistry, Umeå University, 901 87 Umeå, Sweden; ⊥ College of Chemistry, Chemical Engineering and Materials Science, Shandong Normal University, 250014 Jinan, China; # Man-Technology-Environment Research Centre (MTM), Örebro University, 70182 Örebro, Sweden

**Keywords:** uranium retention, schwertmannite, jarosite, incorporation mechanism, X-ray absorption spectroscopy, mineral transformation, HERFD-XANES

## Abstract

Schwertmannite and jarosite are naturally occurring iron (Fe) oxyhydroxysulfates with strong sorption capacities for hexavalent uranium [U­(VI)] in various acidic sulfate-rich environments. These metastable minerals commonly undergo recrystallization, particularly in the presence of dissolved Fe^2+^ [Fe­(II)_aq_], which may influence the fate of associated U­(VI). Here, we quantified molecular-level changes in U repartitioning and speciation when U­(VI)-sorbed schwertmannite and jarosite reacted with Fe­(II)_aq_ under near-neutral and anaerobic conditions over 2 weeks. The results show that Fe­(II)_aq_ additions promoted rapid mineral transformation to goethite via a dissolution–reprecipitation pathway, proceeding (near-completely) for schwertmannite but slowly and incompletely for jarosite. Importantly, even at early transformation stages when goethite likely only started forming on the surface of the transforming minerals, the recrystallization process led to near-complete retention of U, predominantly as U­(VI), within the structure of the neo-formed goethite. Subsequent U reduction to U­(V) increased with time but remained incomplete, even after extensive mineral transformation in the presence of 1–50 mM Fe­(II)_aq_ for 2 weeks. The results demonstrate that Fe­(II)-promoted recrystallization of Fe-oxyhydroxysulfates can rapidly and persistently lock both U­(VI) and U­(V) into chemically stable goethite, with important implications for predicting U behavior and designing remediation strategies in various acidic and U-contaminated environments.

## Graphical abstract

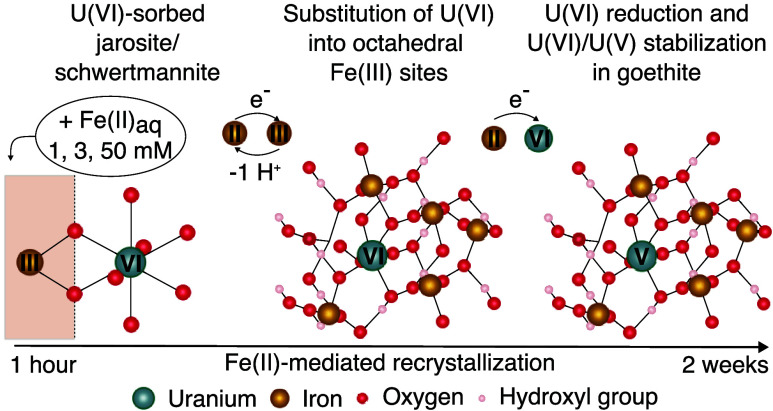

## 1 Introduction

Oxidation of sulfidic soils and rocks, especially those enriched in U (e.g., black shale and waste rocks of U mines), generates abundant acidic and sulfate-rich solutions with elevated U­(VI) concentrations at numerous sites, imposing a long-term threat to surrounding ecosystems.^1–3^ This process also leads to the formation of Fe­(III) oxyhydroxysulfates, primarily as jarosite (KFe_3_(SO_4_)_2_(OH)_6_) and schwertmannite (Fe_8_O_8_­(OH)_8–2x_­(SO_4_)_*x*_·*n*H_2_O, where 1 < *x* < 1.75),^4–6^ which can effectively capture U­(VI) from solution^7–9^ and thereby limit its dispersion and toxicological impacts. However, these minerals are metastable and tend to recrystallize to more stable Fe­(III) oxides under ambient conditions.^7,9–11^ This process can be greatly catalyzed by dissolved Fe^2+^ [Fe­(II)_aq_] under weakly acidic to neutral suboxic conditions,^12,13^ which frequently arise due to waterlogging that favors microbial Fe­(III)-reduction and associated alkalinity production. Such conditions occur during, for example, seasonal water table rise, artificial inundation (sometimes combined with liming), and sediment burial in various acidic environments.^12,14,15^ The natural or human-induced reductive recrystallization of Fe­(III) oxyhydroxysulfates is expected to govern the repartitioning of co-occurring U­(VI), primarily through transformation from surface-sorbed labile species to structurally incorporated and thus more inert fractions. This, in turn, strongly influences the long-term fate and environmental impacts of U in various environments.

Uranium redox behavior in natural systems has commonly been conceptualized as a two-end-member system involving U­(VI) and U­(IV) with contrasting solubilities.^16–18^ However, a growing body of experimental studies has shown that Fe­(II)-promoted reductive recrystallization of Fe­(III) (oxy)­hydroxides under ambient conditions can facilitate the reduction of coexisting U­(VI) to U­(V), which can be subsequently incorporated into the structure of goethite or magnetite, leading to limited exchange with the aqueous phase and thus reduced U mobility,^19–22^ depending on Fe­(II)_aq_ concentrations.^23,24^ While U­(V) is often considered a transient intermediate, structurally incorporated U­(V) into Fe (oxy)­hydroxides, particularly goethite, has been shown to strongly persist even under oxic conditions.^21,25,26^ The persistence is attributed to electron transfer between surface-sorbed Fe­(II) and U­(VI), producing octahedrally coordinated U­(V) that readily incorporates into the lattice of newly formed Fe oxide minerals.^21,27^ Emerging evidence further suggests that a fraction of U­(VI) may also be structurally incorporated into neo-formed goethite and magnetite during Fe­(II)-driven recrystallization of Fe (oxy)­hydroxides.^19,20,23^ However, these previous studies have also shown that elevated Fe­(II) or U loadings may also promote the formation of uraninite,^21,23,24^ which is more prone to reoxidation/remobilization under fluctuating redox conditions^24^ and thus, a less environmentally resilient end product of U reduction. Therefore, it is crucial to elucidate the physicochemical conditions and mechanisms that favor structural incorporation of U­(V) over uraninite formation during reductive recrystallization of Fe­(III) (oxy)­hydroxides. To date, no studies have investigated these processes in systems with Fe­(III) oxyhydroxysulfates, Fe­(II), and U­(VI), and additionally, there is very limited direct identification and quantification of U­(V) in natural minerals under environmentally realistic conditions.

The objective of this study was to determine the reaction pathways and ultimate fate of U when U­(VI)-sorbed schwertmannite and jarosite are reacted with Fe­(II)_aq_ under environmentally relevant conditions. To this end, we conducted two parallel batch experiments over 2 weeks, during which synthetic U­(VI)-sorbed schwertmannite and jarosite were exposed anaerobically to 0–50 mM Fe­(II)_aq_ at pH = 6.5. The experiments were designed to represent near-neutral, Fe-reducing environments that commonly develop following waterlogging of oxyhydroxysulfate-containing sediments in acidic settings. Temporal changes in Fe mineralogy and U speciation were monitored through aqueous-phase analyses, X-ray diffraction (XRD), attenuated total reflectance-Fourier-transform infrared spectroscopy (ATR-FTIR), and synchrotron-based techniques, including Fe and U X-ray absorption spectroscopy (XAS) and high-energy resolution fluorescence detection X-ray absorption near-edge structure (HERFD-XANES) spectroscopy. Our findings provide new mechanistic insights into Fe­(II)-driven U stabilization pathways and inform remediation strategies for U-contaminated acidic and sulfate-rich environments.

## 2 Materials and Methods

### 2.1 Synthesis and Composition of U­(VI)-Sorbed Schwertmannite and Jarosite

U­(VI)-sorbed schwertmannite (USCH) and jarosite (UJAR) were prepared by reacting freshly synthesized jarosite and schwertmannite with approximately 0.21 mM U­(VI) at pH 5.0. To avoid the formation of surface U precipitates (e.g., schoepite), the U stock solution was added incrementally (for details, see Text S1). Our previous U XAS data have shown that these conditions yield exclusively bidentate uranyl surface complexes on both minerals.^7^ The resulting mineral solids were washed with ultrapure water, air-dried, and milled, after which triplicates of the milled materials were fully digested in *aqua regia*. The resulting solutions were analyzed for the concentrations of Fe, K, and U by inductively coupled plasma mass spectrometry (ICP-MS) in order to determine the total contents of metals in the minerals. These were for USCH 442 ± 21 g/kg Fe and 2012 ± 81 mg/kg U, and for UJAR 303 ± 12 g/kg Fe, 77 ± 3.7 g/kg K, and 361 ± 25 mg/kg U.

### 2.2 Batch Experiments

Two parallel batch experiments at ambient temperature were conducted to investigate the interactions of the USCH and UJAR with Fe­(II)_aq_ under anaerobic conditions (O_2_ < 0.5 ppm). All solutions were purged with N_2_ gas overnight before being transferred to an anaerobic chamber. Each experiment involved suspending, in 50 mL tubes, 800 mg of USCH or UJAR mineral powder in 40 mL of deoxygenated 0.1 M NaCl solution, containing 0.05 M MES/MOPS buffer (2-(*N*-morpholino)­ethanesulfonic acid and 3-(*N*-morpholino)­propanesulfonic acid) adjusted to pH 6.5 with dilute NaOH. The suspensions were placed on a vertical tube rotator for 6 h to allow stabilization of the mineral-solution system and conditioning of reactive surface sites before Fe­(II)_aq_ addition. Thereafter, appropriate volumes of fresh deoxygenated Fe­(II)_aq_ stock solutions were added to achieve three initial Fe­(II)_aq_ concentrations (1, 3, and 50 mM) along with a control in which no Fe­(II)_aq_ was added. Following the Fe­(II)_aq_ additions, the suspensions were capped and continuously mixed on the rotator. After 1 h, 2 h, 4 h, 6 h, 1 day, 4 days, and 2 weeks, duplicate tubes of suspensions were withdrawn from each treatment. A small aliquot of suspension from one tube was filtered through a 0.1 μm membrane, and the retained materials on the membrane were dried and stored in an anaerobic chamber for U XAS and HERFD-XANES analyses. The remaining suspension was capped again and, together with the second tube of suspension, centrifuged outside the anaerobic chamber. The supernatant solutions were immediately measured for pH. After that, they were filtered through 0.2 μm syringe filters, acidified with HNO_3_, and measured for total concentrations of Fe, K, and U by ICP-MS. The obtained solid phases were dried in the anaerobic chamber and stored in airtight containers for Fe XAS, XRD, ATR-FTIR, and scanning electron microscope (SEM) analyses/observations. The overview of analytical methods applied to each sample is given in Table S1.

### 2.3 XRD, ATR-FTIR, and SEM-EDS

Mineralogical changes in the solid samples from the two batch experiments were examined by XRD using the X8 PROTEUM system from Bruker AXS (Cu Kα radiation). Samples were scanned over a 2θ range of 10–80°, and the resulting XRD spectra were normalized to the intensity of the most intense peak. As most characteristic diffraction peaks occur within a 2θ of 10–65°, the XRD patterns were evaluated within this range by comparing with previously published standard spectra and/or reference cards from the PowCod database in the QUALX2.0 software.^28^

The evolution of functional groups and chemical bonds on the surfaces of selected solid samples was evaluated by ATR-FTIR using a VERTEX 70v spectrometer (Bruker). Spectra were collected over 600–4000 cm^–1^ at a nominal resolution of 2 cm^–1^, coadding 100 scans per measurement. Data were processed using Blackman–Harris 3-term apodization and Mertz phase correction.

The morphology and elemental composition of selected solid samples were examined using a JEOL JSM-SEM equipped with a JEOL energy-dispersive spectrometry (EDS) system, operated at an accelerating voltage of 15 kV in a low-vacuum mode, and working distance of 10 mm. Uncoated mineral grain aggregate samples were mounted on copper tape, and moisture was removed with a vacuum pump before introduction into the SEM for analysis.

### 2.4 Fe and U XAS

Iron K-edge and U L_3_-edge XAS were used to examine changes in the solid-phase speciation of Fe and U during the two batch experiments. Iron XAS measurements were performed in transmission mode on ground solid samples at the Hard X-ray Microanalysis (HXMA) beamline of the Canadian Light Source (CLS). Uranium XAS measurements were performed on materials retained on filters under cryogenic condition in fluorescence mode at the same beamline with a 32-element Ge detector. During data collection, the beamline was operated in focused mode, employing a Si(111) monochromator together with Rh-coated collimating and toroidal mirrors in the X-ray beam path. Detailed descriptions of sample preparation, experimental setup, and procedures are provided in Text S2. Both U L_3_-edge XANES and extended X-ray absorption fine structure (EXAFS) data were acquired for selected USCH samples, whereas only U L_3_-edge XANES spectra were collected for selected UJAR samples because of their significantly weaker U fluorescence signals.

The XAS data were processed using the Athena software,^29^ following standard procedures (e.g., energy calibration, background subtraction, and normalization). EXAFS spectra were extracted using a cubic spline function after setting E_0_ as the first inflection point of the absorption edge. The speciation of Fe was quantified by linear combination fitting (LCF) analysis using the SIXpack software.^30^ The *k* ^3^-weighted sample EXAFS spectra were reconstructed via linear combinations of the spectra of schwertmannite, jarosite, lepidocrocite, ferrihydrite, and magnetite collected during our previous session at the CLS.^7^ The LCF analysis began with the reference spectrum giving the lowest *R*-factor. An additional reference spectrum was added stepwise if the *R*-factor decreased by >12% relative to the previous fit. The U EXAFS spectra were converted to *R*-space via Fourier transforms (FT) over the *k* range of 3.0–9.2 Å^–1^ using a Hanning window. The resulting spectra for each sample (with *k*-weighting of 1, 2, 3) were fitted simultaneously in *R*-space (*R* = 1–3.84 Å) using the Artemis program^29^ (for details, see Text S3), to obtain structural information about the local environment around the U atoms.

### 2.5 HERFD-XANES

Uranium M_4_-edge HERFD-XANES was used to examine U oxidation states in selected samples of solid materials retained on the membranes. U HERFD-XANES measurements were performed in fluorescence mode at the ROBL BM20 beamline^31^ at ESRF (Grenoble, France). Measurements were performed under a cryostream with a double-crystal Si(111) monochromator and a Johann-type X-ray emission spectrometer (XES).^32,33^ The XES was operated in vertical Rowland geometry and equipped with five spherically bent Si(220) crystal analyzers (1 m bending radius) aligned to the U M_ß_ (M4–N6) emission line, together with a silicon drift detector.

The U M_4_-edge HERFD-XANES data were processed using the PyMca software,^34^ to confirm spectral stability and average the spectra, followed by ackground subtraction (Savitzky–Golay filter) and area normalization . The quantification of U­(IV), U­(V), and U­(VI) fractions was done by Iterative Target Factor Analysis (ITFA).^35^ Detailed descriptions of the measurements and quantitative determination of U oxidation states are provided in Text S4.

## 3 Results

### 3.1 Aqueous Chemistry

Throughout the two batch experiments, the pH values remained stable, fluctuating between 5.9 and 6.4 for USCH (mostly within 6.0–6.1) and 5.7–6.4 for UJAR (mostly within 5.8–6.3) in all treatments (Table S2). In the two control treatments, Fe­(II)_aq_ concentrations remained consistently low (Table S2). In the UJAR control treatment, considerable amounts of K were released into solution, accounting for over 25% of total K by week 2 (Figure 1A). In the 1 and 3 mM Fe­(II)_aq_ treatments, K loss followed almost identical temporal trends, characterized by a pronounced increase between 6 h and 4 days as well as maximum losses (∼75%) at day 4 or week 2. In contrast, these treatments induced increasing U release during the first 6 h, reaching up to ∼1.5 and ∼5%, respectively, of the total U load (Figure 1B). Thereafter, U release/concentrations declined sharply and stabilized at low levels, similar to that in the control. In contrast, despite containing ∼7 times more U than UJAR, USCH released significantly less U into solution (Table S2), accounting for <0.1% of the total U load with slight increases in the 3 mM Fe­(II)_aq_ treatment at 4 and 6 h (Figure 1C). Despite the low levels of U released into solution, there was an overall trend of decreasing release of U (that is, decreasing concentrations in the solution) with increasing Fe­(II) retention in the minerals (Figure S1).

**Figure 1:**
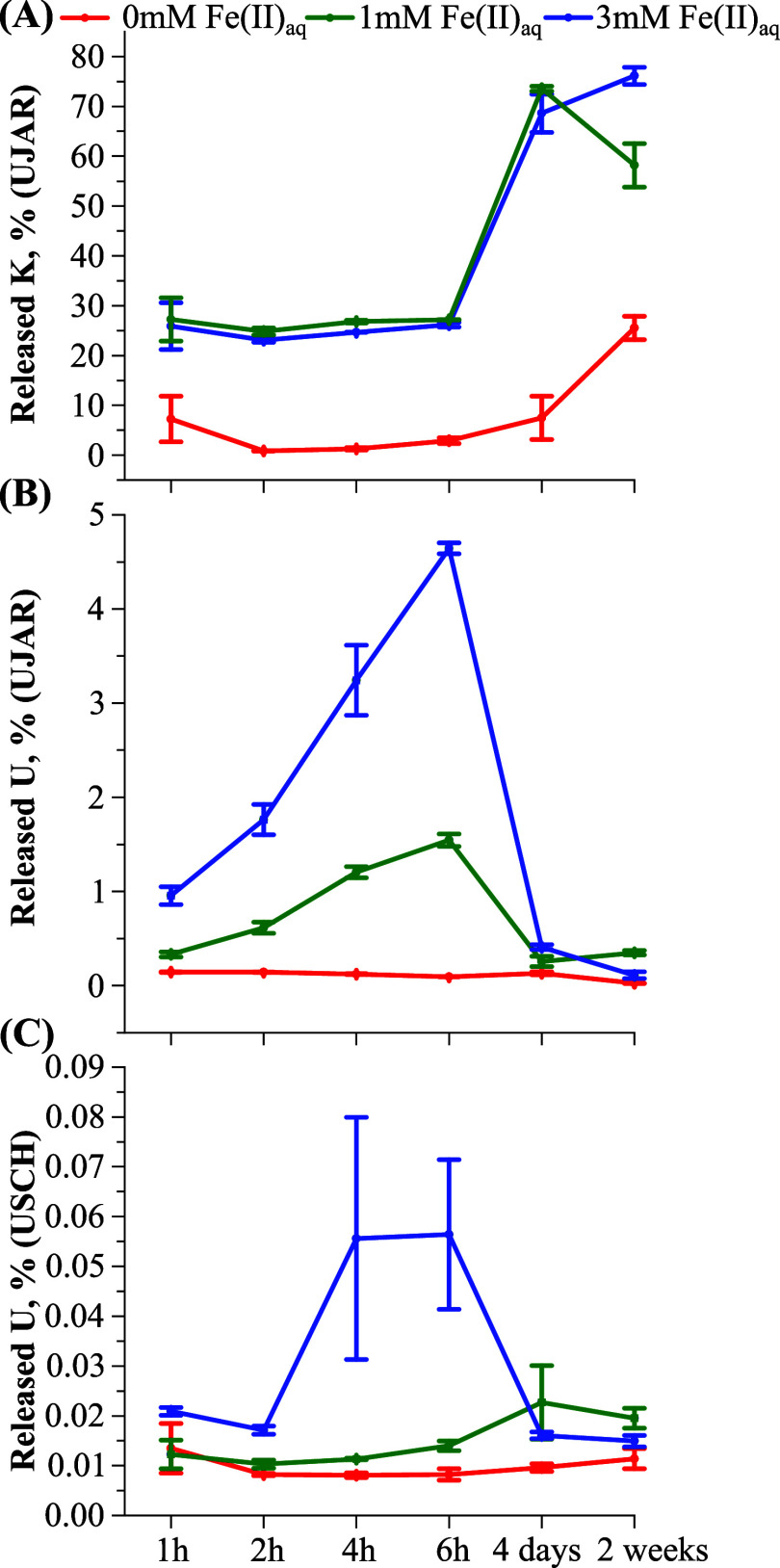
Temporal evolution of total amounts of K and U in solution, presented as percentages relative to their initial amounts in U-sorbed jarosite (UJAR; A, B) and schwertmannite (USCH; C), during reactions with 1 and 3 mM Fe­(II)_aq_ over 2 weeks, compared with the control (with no Fe­(II)_aq_ addition). Data are shown as means ± standard deviations of duplicate samples.

### 3.2 Iron Mineralogical Transformations

The two control samples (at week 2) had XRD peaks characteristic of schwertmannite and K-jarosite, with no detectable peaks from other minerals (Figure 2). Consistently, their ATR-FTIR spectra displayed diagnostic absorption bands of schwertmannite and jarosite (Figure S2, Text S5). Minor deviations in band intensity and position from previous reports^36–39^ likely arose from differences in mineral synthesis procedures.^40,41^ Both control samples also displayed typical morphologies and elemental compositions (e.g., Fe/S ratios) for schwertmannite and K-jarosite (Figures S3, S4 and Table S3). Taken together, these results showed that there was no detectable mineralogical transformation or alteration of the UJAR and USCH during the control treatments.

**Figure 2:**
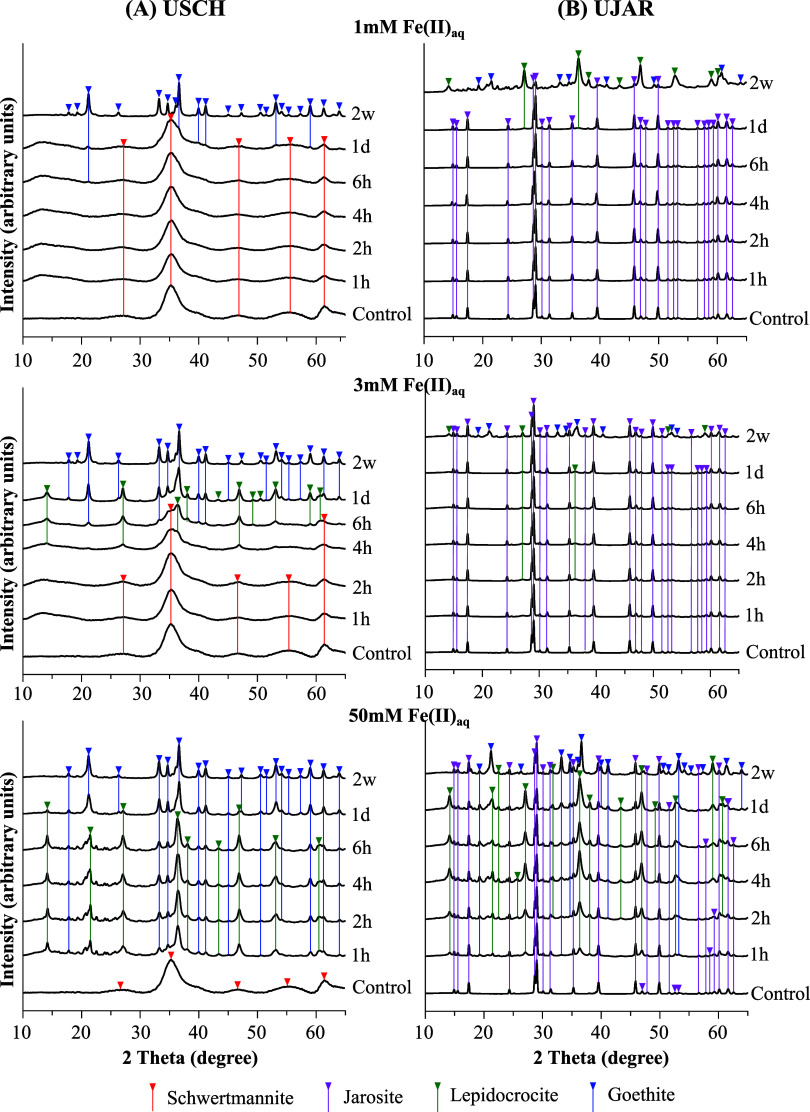
Evolution of XRD patterns for USCH (A) and UJAR (B) during reactions with three levels of Fe­(II)_aq_ over 2 weeks, as compared to the control determined after a 2-week reaction with no Fe­(II)_aq_ addition.

As revealed by the XRD and ATR-FTIR analyses, increasing additions (1–50 mM) of Fe­(II)_aq_ progressively promoted transformation of UJAR or USCH toward goethite over time, with lepidocrocite as an intermediate phase, and with the most pronounced transformation by week 2 (Figure 2). An exception to this general trend of mineral transformation was UJAR reacted with 1 mM Fe­(II)_aq_ at week 2, which exhibited markedly weaker XRD peaks and FTIR bands for jarosite than the corresponding samples from the two high Fe­(II)_aq_ treatments (Figures 2b and S2). This sample was thus an outlier for some reason. Moreover, the transformation of USCH was noticeably faster and more efficient than that of UJAR under identical Fe­(II)_aq_ conditions (Figures 2 and S2). The progressive mineral transformation was in line with the SEM-EDS results, which, although indicative due to small crystal sizes, showed an overall increase over time in the Fe to S ratios for both minerals, along with marked K depletion in UJAR (Table S3). Morphologically, it appears that USCH grains became smoother with fewer fine nodular features, while UJAR particles became increasingly irregular with uneven surfaces (Figures S3 and S4).

Consistent with the XRD and ATR-FTIR results, the LCF-EXAFS analysis of the USCH samples at week 2 predicted that schwertmannite was unaltered in the control treatment but was entirely transformed into goethite in all Fe­(II)_aq_ treatments (Table S4). The pattern was less clear for the corresponding 2-week jarosite samples, for which diagnostic XRD peaks and FTIR bands persisted (Figures 2 and S2), while the LCF-EXAFS analysis predicted that jarosite was nearly completely transformed into lepidocrocite and goethite, with goethite strongly dominating in the high Fe­(II)_aq_ treatments (3 and 50 mM) (Table S4). Given the well-documented high accuracy (within ±3–5%) of the LCF-EXAFS approach for quantifying Fe mineralogical composition^42–44^ and that most of the K was lost from UJAR at week 2 (Figure 1A), there is strong evidence of extensive jarosite transformation at this time despite the persistence of XRD peaks and FTIR bands for this mineral.

### 3.3 Solid-Phase Reduction and Repartitioning of U

The U M_4_-edge HERFD-XANES spectra of UJAR and USCH samples displayed systematic shifts in both peak position and shape with increasing Fe­(II) loadings and reaction time (Figure 3). For the control samples (0 mM Fe­(II)_aq_, 2 weeks), the spectra showed a main peak at ∼3726.5 eV and a distinct high-energy shoulder at ∼3728.7 eV, which are typical of U­(VI), pointing to the strong persistence of surface-sorbed U­(VI) in the absence of Fe­(II) under our experimental conditions. In contrast, the additions of Fe­(II)_aq_, especially at 50 mM, induced progressive spectral changes toward the end of the experiments, including suppression of the distinct U­(VI) shoulder and a shift of the main peak toward lower energies, approaching that of the U­(V) standard.^45^ These spectral changes were more pronounced and developed more rapidly during the USCH experiment. Additionally, no discernible shoulder or subpeak appears on the low-energy side of the main peak, indicating no or negligible contribution from U­(IV) species. Consistent with the spectra features, the ITFA delivered the following results. First, the HERFD-XANES spectra of the Fe­(II)-treated samples were well reproduced by two components, representing U­(VI) and its reduction product U­(V). Second, the fraction of U­(V) increased with increasing Fe­(II) loadings and reaction time, reaching 60 and 71% after 2 weeks in the 50 mM Fe­(II)_aq_ treatments of UJAR and USCH, respectively (Figure 3). A minor exception to this general trend was observed for the 3 mM Fe­(II)_aq_ treatment of USCH, for which the U­(V) fraction at 4 h was slightly lower than at 1 and 2 h (Figure 3A). A more notable exception occurred for USCH reacted with 1 mM Fe­(II)_aq_ at 2 weeks, which yielded a U­(V) fraction exceeding that of the corresponding 2-week sample in the 3 mM Fe­(II)_aq_ treatment (55%), but matching that of the 50 mM Fe­(II)_aq_ treatment (71%) (Figure 3A). The cause of these deviations is unclear but may reflect variability in Fe­(II)-mediated reduction and stabilization of U­(VI), as well as uncertainties associated with the ITFA. Third, the reduction of U­(VI) to U­(V) was more prominent in the USCH experiment (Figure 3).

**Figure 3:**
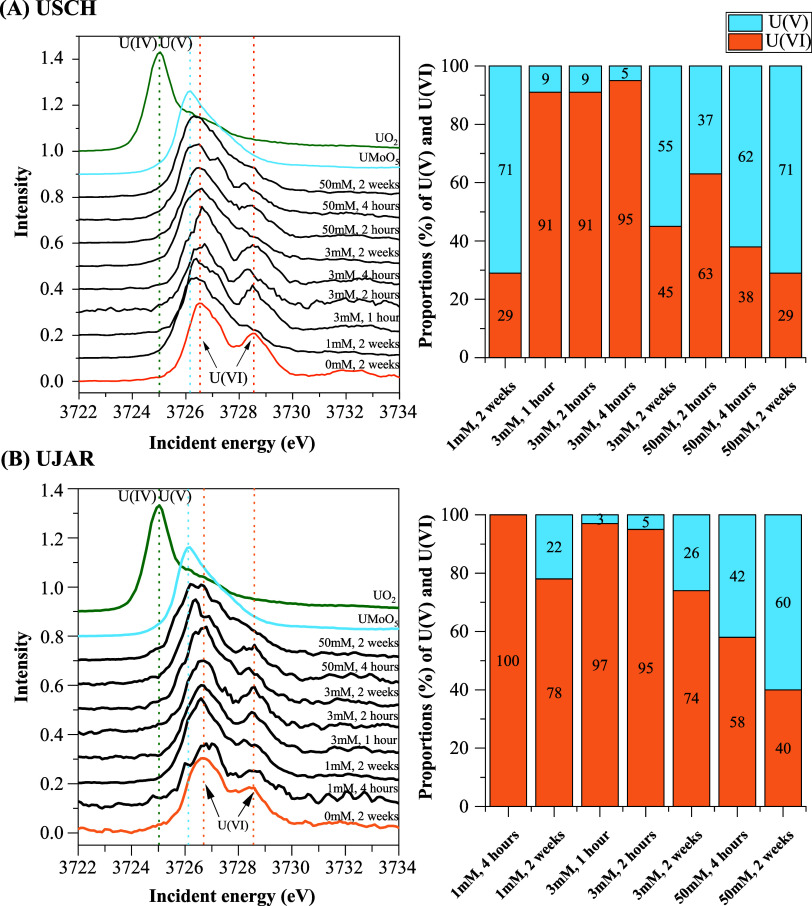
Temporal changes in U M_4_-edge HERFD-XANES spectra and corresponding proportions of U­(V) and U­(VI) (derived from the ITFA) for selected USCH (A) and UJAR (B) samples reacted with different Fe­(II)_aq_ levels over 2 weeks. Spectra were compared with UO_2_, UMoO_5_, and the corresponding 2-week USCH or UJAR control (as U­(VI) references). Dashed lines mark the characteristic peaks for U­(IV), U­(V), and U­(VI) species. The uncertainties of the ITFA-derived U­(V) and U­(VI) proportions were estimated to be within 2%.

The U L_3_-edge XANES spectra of Fe­(II)-treated UJAR and USCH samples also differed markedly from those of uraninite and the UJAR or USCH controls representing U­(VI) standards (Figure 4). Notably, the edge energy of these samples was intermediate between that of uraninite and the U­(VI) standards, while the post-edge shoulder shifted to higher energy relative to the U­(VI) standards. These spectral features have been used as indicators of U­(V).^19,20^ Additionally, the main absorption peak exhibited a clear asymmetric profile (Figure 4), which is also a typical feature of U­(V)-bearing phases^46,47^ and thus provides further evidence for the existence of the intermediate U oxidation state. This feature was particularly relevant for the 1 mM Fe­(II)_aq_ treatment of USCH at 1 and 4 h, for which HERFD-XANES data were not available.

**Figure 4:**
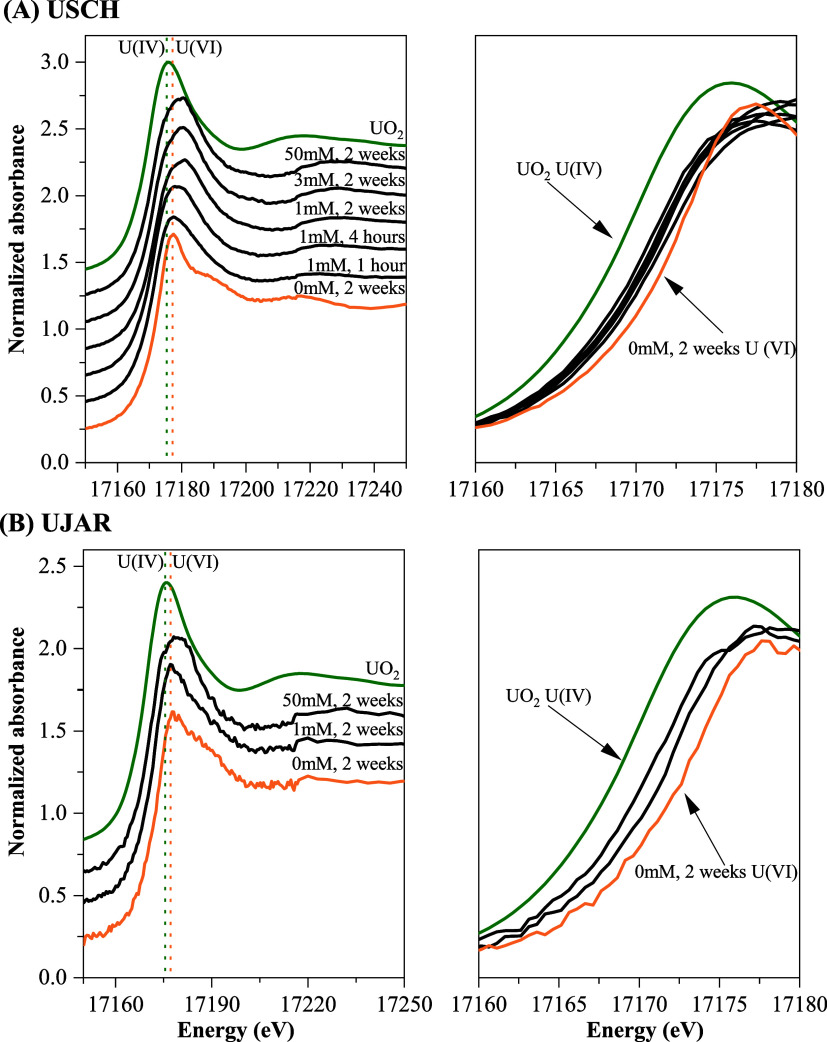
Normalized U L_3_-edge XANES spectra of selected USCH (A) and UJAR (B) samples, compared with those of UO_2_ and the corresponding 2-week USCH or UJAR control (as U­(VI) references). The dashed lines in the left panel mark the positions of the main absorption maxima for U­(IV) and U­(VI). The right panels show enlarged edge regions with spectra overlaid for comparison with U­(IV) and U­(VI) references.

The U L_3_-edge EXAFS spectrum of the USCH control was best fitted with 2.1 ± 0.3 axial O at 1.80 ± 0.02 Å, 7.4 ± 1.5 equatorial O at 2.36 ± 0.04 Å, and 0.3 ± 0.7 Fe atoms at 3.36 ± 0.15 Å (Table 1), consistent with bidentate uranyl surface complexation on schwertmannite.^7^ This showed that U­(VI) remained tightly bound as inner-sphere complexes with Fe sites on schwertmannite in the absence of Fe­(II) under the experimental conditions. In contrast, for the four analyzed samples with Fe­(II)_aq_ additions (1 mM Fe­(II)_aq_ for 4 h and 2 weeks, as well as with 3 and 50 mM Fe­(II)_aq_ for 2 weeks), there was a pronounced yet consistent change in the local U coordination environment, as reflected by distinct U EXAFS spectra and corresponding FT peaks, particularly the marked increase in the magnitude of U–Fe peaks relative to the control (Figure S5). These spectral features were well reproduced using a structural model similar to that of Boland et al.,^19,20^ yielding 0.74–1.48 axial O at 1.72–1.79 Å, 5.0–7.8 equatorial O at 2.04–2.12 Å, plus two U–Fe shells at 3.13–3.17 and 3.50–3.59 Å, respectively (Table 1). The U–O equatorial and U–Fe distances are consistent with those derived from atomistic simulations and experimental studies in which U­(VI/V) substitutes for octahedral Fe­(III) sites in goethite.^19–21,48–50^ These features and results collectively suggest that U in the Fe­(II)-treated USCH samples was predominantly incorporated into the lattice of goethite.

**Table 1:** Uranium L_3_-Edge EXAFS Fitting Results for Selected USCH Samples^a^

	USCH 0 mM Fe(II)_aq_-2 weeks				USCH 1 mM Fe(II)_aq_-4 h			USCH 1 mM Fe(II)_aq_-2 weeks		
Δ*E* (eV)	7.1 (±3.7)			Δ*E* (eV)	–9.6 (±6.3)			–7.3 (±3.3)		
*R*-factor^b^	0.016			*R*-factor^b^	0.040			0.031		
path	CN	*R* (Å)	σ^2^ (Å^2^)	path	CN	*R* (Å)	σ^2^ (Å^2^)	CN	*R* (Å)	σ^2^ (Å^2^)
U–O_ax_	2.06 (±0.28)	1.80 (±0.02)	0.001	U–O_ax_	1.35 (±0.62)	1.79 (±0.03)	0.003^c^	1.48 (±0.38)	1.74 (±0.01)	0.003^c^
U–O_eq_	7.36 (±1.49)	2.36 (±0.04)	0.015	U–O_eq_	5.01 (±1.26)	2.08 (±0.04)	0.012^c^	6.98 (±0.85)	2.05 (±0.02)	0.012^c^
U–Fe	0.30 (±0.71)	3.36 (±0.15)	0.004	U–Fe_1_	0.16 (±0.86)	3.17 (±0.49)	0.007^c^	0.89 (±0.59)	3.13 (±0.04)	0.007^c^
				U–Fe_2_	2.44 (±0.80)	3.53 (±0.05)	0.003^c^	3.21 (±0.47)	3.52 (±0.03)	0.003^c^

	USCH 3 mM Fe(II)_aq_-2 weeks			USCH 50 mM Fe(II)_aq_-2 weeks		
Δ*E* (eV)	–8.1 (±5.4)			0.7 (±2.7)		
*R*-factor^b^	0.008			0.010		
path	CN	*R* (Å)	σ^2^ (Å^2^)	CN	*R* (Å)	σ^2^ (Å^2^)
U–O_ax_	1.43 (±0.69)	1.72 (±0.02)	0.003^c^	0.74 (±0.21)	1.77 (±0.02)	0.003^c^
U–O_eq_	7.81 (±1.60)	2.04 (±0.04)	0.012^c^	6.50 (±0.00)	2.12 (±0.02)	0.012^c^
U–Fe_1_	0.99 (±0.99)	3.14 (±0.07)	0.007^c^	0.73 (±0.39)	3.16 (±0.04)	0.007^c^
U–Fe_2_	3.62 (±0.88)	3.50 (±0.04)	0.003^c^	2.61 (±0.69)	3.59 (±0.03)	0.003^c^

a CN, coordination number; *R*: interatomic distance; σ^2^, Debye–Waller factor; and Δ*E*, energy shift parameter.

b *R*-factor = ∑((data-fit)^2^/∑data^2^).

c Fixed to the values as reported by Boland et al.^20^

## 4 Discussion

### 4.1 Fe­(II)-Catalyzed Dissolution–Reprecipitation Pathway of Jarosite and Schwertmannite Transformation

The XRD and FTIR data did not reveal any noticeable transformations of jarosite or schwertmannite in the 2-week control experiments (Figures 2 and S2), consistent with their slow transformation kinetics under near-neutral pH conditions in the absence of Fe­(II)_aq_.^51–53^ In contrast, increasing Fe­(II)_aq_ additions induced strong and progressive transformation of both minerals to lepidocrocite, which is kinetically more accessible due to its layered structure under nonequilibrium conditions,^37^ and ultimately to goethite (Figure 2 and Table S4). The transformations are explained by the well-established Fe­(II)-catalyzed dissolution–reprecipitation pathway, that is, Fe­(II)_aq_ readily sorbs onto jarosite or schwertmannite surfaces under near-neutral pH, producing Fe­(II) surface complexes that undergo electron transfer and atom exchange (ETAE) with structural Fe­(III).^12,51,52,54^ For jarosite, this mechanism is further supported by the close correspondence between increasing aqueous K^+^ (released from the mineral) and the emergence of lepidocrocite and goethite toward the end of the experiments (Figures 1, 2 and Table S2). Jarosite used for the experiments possesses a roughly 3-fold lower specific surface area than schwertmannite,^7^ and has high crystallinity as indicated by its sharp and well-defined XRD peaks (Figure 2). These properties thus limited Fe­(II) sorption and ETAE efficiency, thereby suppressing the rates and extents of jarosite transformation relative to schwertmannite under identical conditions.

### 4.2 Goethite-Driven Stabilization of Uranium: Structural Incorporation of U­(VI) Followed by Partial Reduction to U­(V)

In both control treatments, there was negligible release of U from the minerals into solution (Figure 1B,C). When Fe­(II)_aq_ was added, the U concentrations in solution increased relative to the controls but remained low, corresponding to less than 5.0% and less than 0.1% of the total U load in the UJAR and USCH samples, respectively (Figure 1). Notably, the released amounts of U overall decreased with increasing Fe­(II) retention in the minerals (Figure S1), suggesting a key role of Fe­(II)_aq_ sorption and subsequent mineral transformation in driving U retention during the two batch experiments.

Reactions of USCH with the three Fe­(II)_aq_ levels over 2 weeks induced pronounced changes in U oxidation state and coordination environment in the solid phase, from initially surface-sorbed U­(VI) to a mixture of structurally incorporated U­(VI) and U­(V) in neo-formed goethite (Figures 3, 4, S5 and Table 1). Notably, the accumulation of U­(V) overall coincided with the extent of Fe­(II)-mediated mineral transformation, particularly the formation of goethite (Figures 2–4 and S3). This temporal relationship is consistent with previous corresponding ferrihydrite-based studies,^19–21^ and shows that U­(VI) reduction during Fe­(II)-promoted mineral transformation occurs on similar time scales as goethite grows. In the 2-week samples, in which only goethite was detected (Figure 2 and Table S4) and the U was structurally incorporated into the goethite lattice as suggested by the U L_3_-edge EXAFS results (Table 1 and Figure S5), U­(V) dominated, but still 29–45% of the U occurred as U­(VI) according to the HERFD-XANES data (Figure 3). This showed that the U reduction during the complete transformation of schwertmannite to goethite was only partial. Lepidocrocite occurred as an intermediate phase during the transformation processes (Figure 2A), but its contribution to U dynamics and stabilization was likely minor, as U­(V) has been shown to preferentially incorporate into goethite even when it is only a minor phase in systems with abundant lepidocrocite formation.^55^ These observations thus collectively suggest that goethite formation represents the critical step governing both U­(VI) reduction and structural stabilization in the USCH systems.

The U L_3_-edge EXAFS results further show that extensive U incorporation into goethite occurred as early as 4 h in the 1 mM Fe­(II)_aq_ treatment of USCH (Table 1 and Figure S5), despite the absence of clearly resolved goethite diffraction peaks at this time point (Figure 2A). The appearance of weak goethite peaks at 6 h suggests that minor amounts of neo-formed goethite likely had formed earlier (Figure 2A), most plausibly within the outermost layers of USCH particles. At similar reaction times but with 3 mM Fe­(II)_aq_, for which HERFD-XANES data were obtained, only 5% of U­(VI) (9% for the shorter reaction times) was reduced to U­(V) (Figure 3A). It is thus reasonable to assume that U in the USCH-1 mM Fe­(II)_aq_ treatment at 4 h, in which structurally incorporated U strongly dominated, experienced only a minimal reduction. These features collectively suggest that, during the early stages of the experiments, U was incorporated into the goethite lattice predominantly in the hexavalent state. This is consistent with the experimental conditions, in which U­(VI) was initially sorbed onto the outermost layers of schwertmannite, where Fe­(II) sorption and subsequent electron transfer to structural Fe­(III) and early goethite nucleation are expected to occur.

Uranium-sorbed schwertmannite that was previously synthesized using the same method as in this study had a specific surface area of 4.1 m^2^/g,^7^ corresponding to an initial U surface loading of ∼1.2 U atoms/nm^2^. This implies that a large fraction of reactive surface sites on USCH was covered by U­(VI). Under the carbonate-free, near-neutral conditions of the experiments, U­(VI) on the surface of USCH was present as positively charged UO_2_^2+^ and UO_2_OH^+^ species.^56^ It is likely that the Fe­(II)_aq_ was preferentially sorbed to surface domains without U­(VI), favoring ETAE between adsorbed Fe­(II) and Fe­(III) within these domains, followed by Fe­(III) hydrolysis and subsequent goethite nucleation. These processes facilitated structural incorporation of adjacent surface-sorbed U­(VI), as evidenced by the limited reduction of U­(VI) without any detectable U­(IV) phases (e.g., uraninite) at early reaction times (Figures 3A, 4A, S5 and Table 1). The structural incorporation likely proceeded via substitution of U­(VI) into octahedral Fe­(III) sites involving edge-sharing Fe­(III) vacancies, which accommodates distorted uranyl-like coordination and, together with hydroxyl deprotonation, provides charge compensation.^23,49,50^ Following structural incorporation, electron transfer from Fe­(II) to incorporated U­(VI) is expected to proceed progressively with time, consistent with increasing fractional amounts of U­(V), most clearly seen from 2 to 4 h and further to 2 weeks in the 50 mM Fe­(II)_aq_ treatment (Figure 3A). However, no quantifiable U­(IV) was observed in any treatments, even at the highest Fe­(II)_aq_ concentration after 2 weeks, indicating that further reduction of structurally incorporated U was infeasible. This supports previous density functional theory calculations showing that incorporation of U­(IV), owing to its large ionic radius, would induce substantial lattice distortion in goethite and is therefore energetically unfavorable.^57^ The persistence of a substantial U­(VI) fraction, even after (near-)­complete mineral transformation to goethite, indicates that electron transfer to structurally incorporated U­(VI) becomes increasingly unfavorable or kinetically constrained. While mixed U­(VI)/U­(V) phases in neo-formed goethite have been inferred previously from EXAFS-derived axial oxygen contributions^19,20^ or U–O bond lengths,^23^ our HERFD-XANES data, combined with U EXAFS results, provide the first direct experimental evidence of this feature, suggesting that partial U reduction is not a prerequisite for U incorporation into goethite as proposed previously.^21^

Our results also suggest that similar mechanisms governed U repartitioning during Fe­(II)-promoted transformation of UJAR. That is, there was an overall correspondence between U­(V) accumulation and goethite formation (Figures 2–4) and a strong decline in dissolved U concentrations following the onset of extensive jarosite transformation after 6 h (Figure 1B), indicating early-stage structural U incorporation into neo-formed goethite. The consistently lower proportions of U­(V) in the UJAR than USCH treatments under similar conditions (Figure 3) were in line with the slower and less complete transformation of jarosite to goethite (Figure 2 and Table S4). For example, in the 1 and 3 mM Fe­(II)_aq_ treatments, jarosite, together with substantial amounts of lepidocrocite, still persisted after 2 weeks (Table S4). Overall, the results demonstrate that Fe­(II)-promoted transformation of both USCH and UJAR efficiently stabilizes U through structural incorporation into neo-formed goethite, with reduction to U­(V) occurring subsequently and remaining incomplete even with extensive or full mineral transformation to goethite.

### 4.3 Environmental Implications

In a variety of acidic and sulfate-rich environments, in which jarosite and schwertmannite are typically abundant, there are zones or horizons where waterlogging is substantial over shorter or longer spells, favoring microbial Fe­(III) reduction and subsequent generation of Fe­(II)_aq_ as well as alkalinity that increases the pH. Under such conditions, Fe­(III) oxyhydroxysulfates can undergo intensive Fe­(II)-promoted transformation to goethite, thereby influencing the fate of U initially sorbed on the oxyhydroxysulfates. Hence, the results and findings of this study, which explicitly target these conditions, are broadly relevant to improving our understanding of the behavior and fate of U in these environmental systems.

A key outcome of this work is the demonstration that Fe­(II)-promoted recrystallization of U­(VI)-bearing schwertmannite and jarosite can rapidly and near-quantitatively redistribute initially surface-sorbed U­(VI) (up to ∼0.2 wt %) to structurally incorporated U­(V) and U­(VI) within neo-formed goethite, with no detectable formation of discrete U­(IV) phases (e.g., uraninite). Previous studies have shown that such incorporation stabilizes U chemically and thus makes it more resistant to remobilization during subsequent redox fluctuations (e.g., exposure to oxic, Fe-reducing, or even sulfidic conditions), as compared to surface-adsorbed U­(VI) or uraninite.^24,49,58^ Given that goethite has a high thermodynamic stability in the oxic environment and can also persist under reducing conditions, the near-quantitative redistribution of schwertmannite- and jarosite-sorbed U­(VI) into the structure of goethite as U­(V)/U­(VI) represents an efficient and long-term U attenuation pathway. From a remediation perspective in terms of U dispersion, it is thus beneficial to promote and sustain Fe-reducing yet nonsulfidic conditions in Fe­(III) oxyhydroxysulfate-rich systems. Such conditions favor the generation of Fe­(II)_aq_ and subsequent Fe­(II)-promoted transformation of Fe­(III) oxyhydroxysulfates to goethite, thereby facilitating structural incorporation of U while minimizing secondary phase transformation of goethite (e.g., via sulfidation) that would remobilize the U. Thus, incorporating these Fe­(II)-driven transformation pathways into conceptual geochemical models is desirable and will improve our ability to develop and construct more effective remediation strategies for many U-contaminated and sulfate-rich environments. In particular, focus should be on maintaining Fe-reducing yet nonsulfidic conditions via, e.g., controlling water-level or inundation dynamics.

Finally, our experiments demonstrate that goethite can accommodate substantial amounts of structurally incorporated U­(V), both in absolute and fractional terms (up to ∼0.14 wt % and ∼71% of the total U pool). Given the widespread occurrence and high stability of goethite, these results indicate that structurally stabilized U­(V) may represent a significant and long-lived U pool in many natural environments. Considering the scarcity of HERFD-XANES data on U­(V) in goethite, our findings also hold important implications for the interpretation of U geochemistry and isotopic signatures in natural goethite, and associated geological events and (bio)­geochemical processes.

## Supplementary Material



## Notes

Detailed information on the preparation of U­(VI)-sorbed jarosite and schwertmannite, Fe and U XAS data collection, U L_3_-edge EXAFS fitting, HERFD-XANES measurements, and quantification of U oxidation states, interpretation of FTIR spectra plus additional tables/figures showing a summary of all analytical methods, evolution of water chemistry, solid-phase Fe speciation, EDS results, released U vs retained Fe­(II), ATR-FTIR spectra, U EXAFS spectra and modeled fits, and SEM images (PDF)
