# Supplementary material for: Stabilization of U(V) and U(VI) in Goethite Formed by Recrystallization of Fe-Oxyhydroxysulfates
Source: Environ Sci Technol. 2026 May 19;60(21):15299–309. doi: 10.1021/acs.est.6c02403 (PMC13235564; doi:10.1021/acs.est.6c02403)
Supplement: Supplementary file 1 [file es6c02403_si_001.pdf]

Supporting information (SI) for

Stabilization of U(V) and U(VI) in Goethite Formed by Recrystallization of Fe-Oxyhydroxysulfates

Liubov Kononova[a], Mats Åström[a], Elena F. Bazarkina[b,c], Damien Prieur[b,c], Kristina O. Kvashnina[b,c], Tao Luo[d,e], Jean-François Boily[d], Henrik Drake[a], Viktor Sjöberg[f], Changxun Yu[a*]

[a]Centre for the Environment (CENWIN), Linnaeus University, 39231 Kalmar, Sweden

[b]The Rossendorf Beamline (BM20), The European Synchrotron, 38043 Grenoble, France

[c]Institute of Resource Ecology, Helmholtz-Zentrum Dresden-Rossendorf, 01328 Dresden, Germany

[d]Department of Chemistry, Umeå University, 901 87 Umeå, Sweden

[e]College of Chemistry, Chemical Engineering and Materials Science, Shandong Normal University, 250014 Jinan, China

[f] Man-Technology-Environment Research Centre (MTM), Örebro University, 70182 Örebro, Sweden

[*]**Corresponding author: yuchangxun2006@163.com; changxun.yu@lnu.se**

Number of pages (15)

Number of Figures (6)

Number of tables (4)

Text S1. Preparation of U(VI)-sorbed jarosite and schwertmannite

Jarosite was prepared according to Baron and Palmer, 1996[1], by dissolving KOH and $Fe_2(SO_4)_3$ in deionized water at 95 °C. The suspension was continuously stirred on a hot plate. After 4 h, the precipitates were allowed to settle and the supernatant solution (pH=~1.6) was decanted. The resulting precipitates were then washed with deionized water, dried at 110°C for 24 h, and pulverized using a mill.

Schwertmannite was synthesized according to Regenspurg et al, 2004[2]. Briefly, $FeSO_4 \cdot 7H_2O$ was dissolved in deionized water and reacted with H_2O_2 (30%) under continuous stirring. The resulting suspension was allowed to settle. The final pH was 2.2. The precipitates were rinsed with MQ water, air-dried, and then ground to fine powder using a mill.

Finally, U(VI)-sorbed jarosite (UJAR) and schwertmannite (USCH) were prepared by resuspending jarosite and schwertmannite powder in 2 L of deionized water, respectively. The pH of each suspension was adjusted to 5 with diluted NaOH. When the pH stabilized, 5 mL 85 mM U stock solution was added incrementally to each of the two suspensions. The suspensions were vigorously stirred for 12 h, during which the pH was regularly checked and readjusted if needed. After that, the mineral particles were obtained via centrifugation and repeatedly washed with deionized water to remove excess U species, before being oven-dried at 40 ℃ overnight. The dry minerals were finely pulverized using an agate mortar.

Text S2. Iron and U XAS data collection

Iron K-edge and U L_3-edge XAS measurements were performed at the Hard X-ray Microanalysis (HXMA) beamline at the Canadian Light Source. During the measurements, the beamline was configured under its focused operation mode with monochromator Si (1 1 1) crystal with Rh collimating and toroidal mirrors in the X-ray beam path. The XAS data at the Fe K-edge were collected in transmission mode at room temperature, while those at the U L_3-edge in fluorescence mode using a 32-element Ge solid-state detector under cryogenic condition. For Fe XAS analyses, powder samples were diluted with polyethylene and packed into Teflon holders sealed on both sides with Kapton tape. U XAS measurements were performed on solids retained on membrane filters, which were cut and sealed between layers of parafilm and Kapton tape inside an anaerobic chamber (O_2 <0.5 ppm). All prepared samples were transported in N_2-filled gas-tight containers. Energy calibration was performed by simultaneously recording the XAS spectrum of a Fe and Y foil, respectively. The scan sizes

for the pre-edge, XANES, and EXAFS regions were 10 eV/step, 0.5 eV/step, and 0.05 $Å^{-1}$/step, respectively. To improve signal-to-noise ratios, 2 scans at the Fe K-edge and 4-11 scans at the U-L_3 edge were recorded. To minimize possible radiation-induced damage during U XAS measurements, each scan was collected from a fresh spot by moving the X-ray beam between each scan.

Text S3. Uranium L_3-edge EXAFS fitting

For obtaining quantitative structural information on the local environment of around U atoms in USCH samples, k^3-weighted EXAFS spectra (k=3.0 to 9.2 $Å^{-1}$) were fitted in R-space (R=1-3.84 Å) using the Artemis software package. Theoretical scattering amplitudes and phase shifts were generated with FEFF7 using structural models based on: (i) sodium uranyl triacetate[3] (U-O_{ax} and U-O_{eq}), (ii) schwertmannite[4] with U substituting for one Fe site (Fe-U), and (iii) goethite with U substituting for one Fe site (U-Fe_1 and U-Fe_2). For the EXAFS fitting, the amplitude reduction factor was fixed at 1.0, and the energy shift was fitted as a global parameter across all paths. Bond distances and coordination number were floated, while the Debye-Waller factor of U-O_{ax}, U-O_{eq}, U-Fe_1, and U-Fe_2 paths was fixed to 0.003 $Å^2$, 0.012 $Å^2$, 0.007 $Å^2$, and 0.003 $Å^2$, respectively, as reported previously[5].

Text S4. HERFD-XANES measurements at U M_4-edge and quantification of U oxidation states

High-energy resolution fluorescence detected X-ray absorption near edge structure (HERFD-XANES) data at the U M_4-edge were collected on the bending magnet ROBL BM20 beamline of the European Synchrotron Radiation Facility (ESRF, Grenoble, France) [6]. The details about HERFD-XANES measurements and their advantages for the environmental sciences have been given previously. In an anaerobic chamber, the solid samples for the HERFD-XANES measurements were obtained by filtering small aliquots of mineral suspensions through 0.1 µm membranes, which were then dried, cut, and sealed by two layers of Kapton foil (13 microns) in the holders designed for cryogenic measurements. The sample specimens were packed in zip-locks and stored in N_2-filled falcon tubes that were stored in an anoxic container. The container was transported to the ESRF on dry ice. The measurements at the beamline were performed under cryogenic conditions, with the sample cooled using a nitrogen cryostream.

The storage ring was operated at 6 GeV with a ~200 mA current in 7/8+1 filling mode. The energy of the incoming beam was selected using a double Si(111) crystal monochromator and two Rh mirrors operating in the total reflection mode. The beam size was 50 (vertical) × 2000 (horizontal) μm^2. Energy calibration was done using UO_2 reference material, setting the maximum of the main HERFD-XANES peak to 3725 eV. For this study, a Johann-type X-ray emission spectrometer (XES) [7] in a vertical Rowland geometry was equipped with 5 spherically bent crystal analyzers Si(220) with a 1 m bending radius, and a silicon drift X-ray detector (©Ketek). Each Si(220) crystal analyzer was aligned at the maximum of the U M_{β} (M4-N6, i.e. 3d-4f transition) emission line (3339.8 eV) with 75° Bragg angle. The maximum of the corresponding non-resonant emission line was chosen using UO_2 reference. A helium-filled bag was placed to fill the optical path sample-crystal analyzers-detector to minimize the absorption of the fluorescence signal by air. A total experimental energy resolution was estimated to be ~0.7 eV. The U M_4-edge HERFD-XANES spectra were recorded with 0.1 eV step in the range of 3721-3736eV of the incident energy, and the counting time was 3-5 sec per point. Multiple (3-9) HERFD-XANES spectra were recorded for each sample in order to increase signal-to-noise ratios. The X-ray beam at the ROBL beamline has a relatively moderate flux and is horizontally unfocused, which helps to minimize both heat load and eventual X-ray-induced chemical changes. The homogeneity of samples and the absence of beam damage were approved by the comparison of multiple scans measured in the same spot (no evolution with time), fast scans in fresh spots (0.5 sec per point), and the time scans (at the incident energy fixed at 3728.5 eV). All tests showed good reproducibility and no changes with time.

The individual HERFD-XANES spectra were first compared in the PyMca software[8]. After check of the stable spectral shape (i.e. no changes between spectra collected in the same spot), the spectra were averaged, and the background was subtracted using Savitzky-Golay filter with width of 1, the interpolating polynomial degree of 1 and the derivative order of 0. Comparison between a real averaged HERFD-XANES spectrum and a background-subtracted version is shown on the Figure S6. After this, the spectra were normalized to area for the energy range 3721.0-3726.0 eV. The quantification of U(IV), U(V) and U(VI) fractions in each sample was done using Iterative Target Factor Analysis (ITFA), following the procedure of Rosseberg et al. (2003)[9]. HERFD-XANES spectra of UO_2, $UMoO_5$[10], and UJA-0 mM Fe(II)$_{aq}$-2week or USCH-0 mM Fe(II)$_{aq}$-2 week were included as pure U(IV), U(V), and U(VI) compounds, respectively.

Text S5. Interpretation of ATR-FTIR spectra

The ATR-FTIR spectra of the 2-week control samples displayed characteristic bands of their respective starting minerals (Figure S2). For schwertmannite (USCH control), diagnostic features included a broad O-H stretching envelope centered near 3400 cm^{-1} and split $\nu_3(SO_4)$ modes at ~1115 and ~1055 cm^{-1} arising from sulfate in structural tunnels and surface sites. For jarosite (UJAR control), the spectrum exhibited sharper sulfate bands at ~1190, ~1085, and ~1005 cm^{-1}, reflecting the C_{3v} site symmetry of structural sulfate. Both minerals showed H-O-H bending of adsorbed water near 1630 cm^{-1}.

Upon Fe(II) addition, progressive transformation to goethite was evidenced by the emergence of its diagnostic spectral features: a prominent O-H stretch at ~3140 cm^{-1}, Fe-O-H bending modes at ~890 cm^{-1} (in-plane, δ) and ~795 cm^{-1} (out-of-plane, γ), and Fe-O stretching near 630 cm^{-1}. These goethite signatures developed more rapidly and intensely in the USCH system, consistent with its faster transformation kinetics observed by XRD. In the UJAR system, a band at ~1021 cm^{-1} appeared transiently in the 1-hour spectra of the 1 and 3 mM $Fe(II)_{aq}$ treatments, attributable to the diagnostic Fe-O-H bending mode of lepidocrocite, consistent with its detection as an intermediate phase by XRD. Concurrently, the sulfate bands diminished in intensity with increasing Fe(II) concentration and reaction time, reflecting sulfate release during dissolution-reprecipitation.

Table S1. Analytical methods applied to solution and solid samples from the two batch experiments with USCH and UJAR

	ICP-MS	XRD	FTIR	SEM-EDS	Fe K-edge EXAFS	U L_3-edge XANES	U L_3 -edge EXAFS	U M_4-edge HERFD-XANES
**USCH**								
0 mM $Fe(II)_{aq}$, 1 h	X							
0 mM $Fe(II)_{aq}$, 2 h	X							
0 mM $Fe(II)_{aq}$, 4 h	X							
0 mM $Fe(II)_{aq}$, 6 h	X							
0 mM $Fe(II)_{aq}$, 1 day								
0 mM $Fe(II)_{aq}$, 4 days	X							
0 mM $Fe(II)_{aq}$, 2 weeks	X	X	X	X	X	X	X	X
1 mM $Fe(II)_{aq}$, 1 h	X	X	X	X		X		

1 mM Fe(II)$_{aq}$, 2 h	X	X						
1 mM Fe(II)$_{aq}$, 4 h	X	X		X		X	X	
1 mM Fe(II)$_{aq}$, 6 h	X	X						
1 mM Fe(II)$_{aq}$, 1 day		X						
1 mM Fe(II)$_{aq}$, 4 days	X							
1 mM Fe(II)$_{aq}$, 2 weeks	X	X	X	X	X	X	X	X
3 mM Fe(II)$_{aq}$, 1 h	X	X	X					X
3 mM Fe(II)$_{aq}$, 2 h	X	X		X				X
3 mM Fe(II)$_{aq}$, 4 h	X	X						X
3 mM Fe(II)$_{aq}$, 6 h	X	X		X				
3 mM Fe(II)$_{aq}$, 1 day		X						
3 mM Fe(II)$_{aq}$, 4 days	X							
3 mM Fe(II)$_{aq}$, 2 weeks	X	X	X	X	X	X	X	X
50 mM Fe(II)$_{aq}$, 1 h		X	X					
50 mM Fe(II)$_{aq}$, 2 h		X		X				X
50 mM Fe(II)$_{aq}$, 4 h		X		X				X
50 mM Fe(II)$_{aq}$, 6 h		X						
50 mM Fe(II)$_{aq}$, 1 day		X						
50 mM Fe(II)$_{aq}$, 4 days								
50 mM Fe(II)$_{aq}$, 2 weeks		X	X	X	X	X	X	X
**UJAR**								
0 mM Fe(II)$_{aq}$, 1 h	X							
0 mM Fe(II)$_{aq}$, 2 h	X							
0 mM Fe(II)$_{aq}$, 4 h	X							
0 mM Fe(II)$_{aq}$, 6 h	X							
0 mM Fe(II)$_{aq}$, 1 day								
0 mM Fe(II)$_{aq}$, 4 days	X							
0 mM Fe(II)$_{aq}$, 2 weeks	X	X	X	X		X		X
1 mM Fe(II)$_{aq}$, 1 h	X	X	X					
1 mM Fe(II)$_{aq}$, 2 h	X	X						
1 mM Fe(II)$_{aq}$, 4 h	X	X		X				X
1 mM Fe(II)$_{aq}$, 6 h	X	X						
1 mM Fe(II)$_{aq}$, 1 day		X		X				

1 mM $Fe(II)_{aq}$, 4 days	X							
1 mM $Fe(II)_{aq}$, 2 weeks	X	X	X	X	X	X		X
3 mM $Fe(II)_{aq}$, 1 h	X	X	X					X
3 mM $Fe(II)_{aq}$, 2 h	X	X						X
3 mM $Fe(II)_{aq}$, 4 h	X	X		X				
3 mM $Fe(II)_{aq}$, 6 h	X	X						
3 mM $Fe(II)_{aq}$, 1 day		X		X				
3 mM $Fe(II)_{aq}$, 4 days	X							
3 mM $Fe(II)_{aq}$, 2 weeks	X	X	X	X	X			X
50 mM $Fe(II)_{aq}$, 1 h		X	X					
50 mM $Fe(II)_{aq}$, 2 h		X		X				
50 mM $Fe(II)_{aq}$, 4 h		X		X				X
50 mM $Fe(II)_{aq}$, 6 h		X						
50 mM $Fe(II)_{aq}$, 1 day		X						
50 mM $Fe(II)_{aq}$, 4 days								
50 mM $Fe(II)_{aq}$, 2 weeks		X	X	X	X	X		X

Table S2. Temporal changes in dissolved K, Fe, and U concentrations and pH during the two batch experiments with USCH and UJAR

USCH			
	Fe (mg/L)		
	0 mM $Fe(II)_{aq}$	1 mM $Fe(II)_{aq}$	3 mM $Fe(II)_{aq}$
1 h	0.20 (0.18-0.22)	18 (14-22)	62 (61-63)
2 h	0.14 (0.12-0.17)	18 (17-19)	59 (53-65)
4 h	0.28 (0.28-0.29)	13 (12-13)	62 (62-63)
6 h	0.24 (0.23-0.25)	21 (16-26)	70 (61-78)
4 days	0.90 (0.77-1.02)	24 (23-25)	52 (44-59)
2 weeks	0.67 (0.63-0.70)	21 (21-21)	29 (27-30)
	U (µg/L)		
	0 mM $Fe(II)_{aq}$	1 mM $Fe(II)_{aq}$	3 mM $Fe(II)_{aq}$
1 h	5.4 (3.4-7.4)	4.9 (3.8-6.1)	8.4 (8.1-8.7)

2 h	3.3 (3.2-3.4)	4.2 (3.8-4.5)	6.9 (6.5-7.2)	
4 h	3.3 (3.1-3.5)	4.6 (4.5-4.6)	22 (13-32)	
6 h	3.3 (2.8-3.8)	5.6 (5.2-6.0)	23 (17-29)	
4 days	3.9 (3.6-4.2)	9.1 (6.2-12)	6.5 (6.2-6.7)	
2 weeks	4.6 (3.8-5.4)	7.8 (7.0-8.7)	6.0 (5.5-6.5)	
	pH			
	0 mM $Fe(II)_{aq}$	1 mM $Fe(II)_{aq}$	3 mM $Fe(II)_{aq}$	50 mM $Fe(II)_{aq}$
1 h	6.0	6.2	6.0	6.0
2 h	6.0	6.2	6.0	6.0
4 h	6.0	6.1	6.0	6.0
6 h	6.0	6.1	6.4	6.0
4 days	6.1	6.1	6.0	6.4
2 weeks	6.1	6.0	6.0	5.9
**UJAR**				
	K (mg/L)			
	0 mM $Fe(II)_{aq}$	1 mM $Fe(II)_{aq}$	3 mM $Fe(II)_{aq}$	
1 h	111 (41-181)	398 (326-470)	418 (352-485)	
2 h	13 (12-14)	355 (348-362)	382 (371-392)	
4 h	19 (16-23)	379 (379-379)	412 (408-416)	
6 h	45 (35-53)	402 (395-410)	418 (417-418)	
4 days	115 (49-182)	1054 (995-1113)	1130 (1122-1137)	
2 weeks	392 (356-428)	1169 (1143-1195)	894 (826-961)	
	Fe (mg/L)			
	0 mM $Fe(II)_{aq}$	1 mM $Fe(II)_{aq}$	3 mM $Fe(II)_{aq}$	
1 h	0.23 (0.16-0.30)	40 (39-41)	126 (126-126)	
2 h	0.15 (0.14-0.16)	39 (38-39)	125 (125-125)	
4 h	0.23 (0.15-0.30)	41 (40-41)	126 (126-129)	
6 h	0.15 (0.13-0.17)	38 (38-39)	121 (114-127)	
4 days	0.23 (0.15-0.30)	31 (22-41)	88 (87-88)	
2 weeks	0.19 (0.17-0.20)	25 (25-26)	43 (34-52)	
	U (µg/L)			
	0 mM $Fe(II)_{aq}$	1 mM $Fe(II)_{aq}$	3 mM $Fe(II)_{aq}$	

1 h	10 (10-11)	24 (22-26)	68 (62-76)	
2 h	10 (10-10)	44 (40-48)	127 (115-139)	
4 h	8.7 (8.4-9.1)	87 (82-91)	233 (207-260)	
6 h	6.8 (6.8-6.8)	111 (106-116)	334 (330-338)	
4 days	9.4 (8.3-10)	18 (14-22)	29 (27-31)	
2 weeks	1.7 (1.5-1.9)	25 (23-27)	7.8 (5.3-10)	
	pH			
	0 mM $Fe(II)_{aq}$	1 mM $Fe(II)_{aq}$	3 mM $Fe(II)_{aq}$	50 mM $Fe(II)_{aq}$
1 h	6.3	5.8	5.8	5.9
2 h	6.3	5.8	6.2	5.8
4 h	6.3	5.8	5.8	5.8
6 h	6.3	5.8	5.7	6.3
4 days	6.2	5.9	6.3	5.8
2 weeks	6.4	6.2	5.8	5.8

Table S3. SEM-EDS results for selected spots in selected UJAR and USCH samples

**UJAR**	Spot	Fe/S ratios	K contents, atomic %	**USCH**	Spot	Fe/S ratios
0 mM $Fe(II)_{aq}$, 2 weeks	1	2.5	2.5±0.04	0 mM $Fe(II)_{aq}$, 2 weeks	1	6.7
	2	4.4	4.6±0.08		2	8.3
1 mM $Fe(II)_{aq}$, 4 h	1	2.2	5.5±0.06		3	7.8
	2	2.9	4.5±0.06	1 mM $Fe(II)_{aq}$, 1 h	1	6.9
1 mM $Fe(II)_{aq}$, 1 day	1	1.3	3.6±0.04		2	6.9
	2	1.5	3.6±0.05	1 mM $Fe(II)_{aq}$, 4 h	1	6.4
1 mM $Fe(II)_{aq}$, 2 weeks	1	5.5	0.5±0.03		2	7.2
	2	2.9	0.4±0.02	1 mM $Fe(II)_{aq}$, 2 weeks	1	12.4
3 mM $Fe(II)_{aq}$, 4 h	1	1.7	5.6±0.07		2	20.3
	2	1.3	3.7±0.05	3 mM $Fe(II)_{aq}$, 2 h	1	7.6
	3	1.5	4.2±0.05		2	6.2
3 mM $Fe(II)_{aq}$, 1 day	1	1.9	3.6±0.04	3 mM $Fe(II)_{aq}$, 6 h	1	9.0
	2	2.1	3.3±0.03		2	8.5
3 mM $Fe(II)_{aq}$, 2 weeks	1	2.6	2.6±0.04	3 mM $Fe(II)_{aq}$, 2 weeks	1	13.6
	2	2.9	3.6±0.05		2	28.2

	3	3.7	1.9±0.03	50 mM $Fe(II)_{aq}$, 2 h	1	10.3
50 mM $Fe(II)_{aq}$, 2 h	1	1.4	2.2±0.04		2	9.8
	2	3.7	2.2±0.04	50 mM $Fe(II)_{aq}$, 4 h	1	7.5
50 mM $Fe(II)_{aq}$, 4 h	1	3.1	1.2±0.03		2	8.2
	2	2.7	3.6±0.07	50 mM $Fe(II)_{aq}$, 2 weeks	1	10.8
50 mM $Fe(II)_{aq}$, 2 weeks	1	9.9	0.3±0.02		2	7.7
	2	5.3	0.5±0.02			

Table S4. Iron speciation in selected UJAR and USCH samples, quantified by Linear Combination Fitting of k^3-weighted Fe EXAFS Spectra (k = 2-12 Å^{-1}). The component sums were normalized to 100% (initial range was 86−100.2%).

UJAR				
	Goethite, %	Lepidocrocite, %	Jarosite, %	R-factor
1 mM $Fe(II)_{aq}$-2 weeks	49.6	49.5	0.9	0.123
3 mM $Fe(II)_{aq}$-2 weeks	77.4	16.7	5.9	0.057
50 mM $Fe(II)_{aq}$-2 weeks	89.6		10.4	0.039
USCH				
	Goethite, %	Schwertmannite, %	R-factor	
0 mM $Fe(II)_{aq}$-2 weeks		100	0.112	
1 mM $Fe(II)_{aq}$-2 weeks	100		0.064	
3 mM $Fe(II)_{aq}$-2 weeks	100		0.091	
50 mM $Fe(II)_{aq}$-2 weeks	100		0.193	

R-factor = $\Sigma((\text{data-fit})^2/\Sigma \text{data}^2)$.

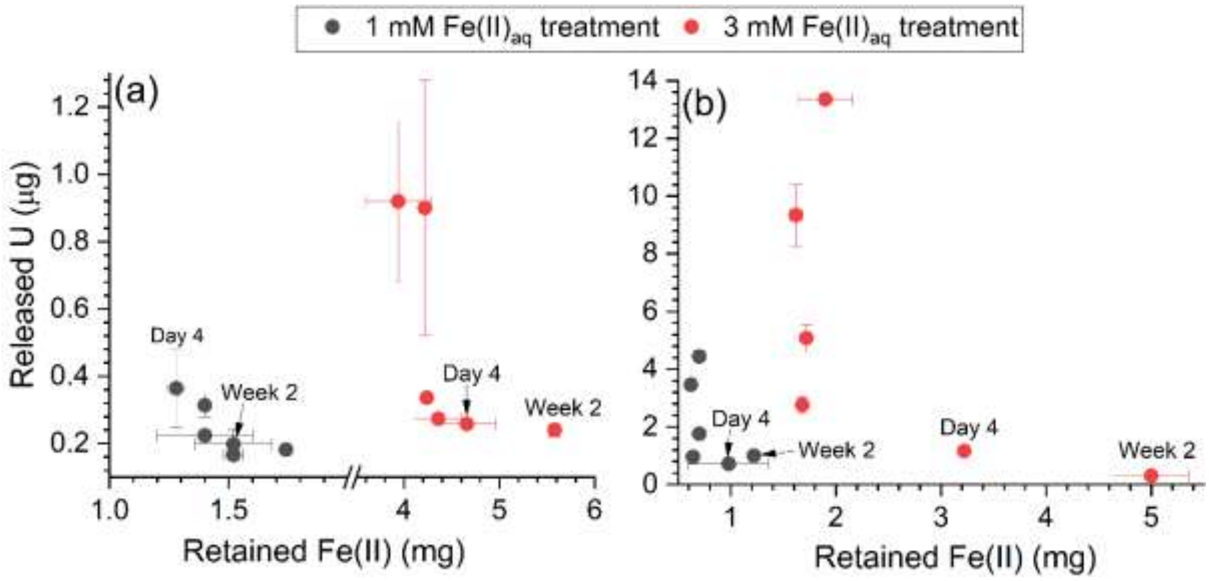

Figure S1. Total U released from the minerals into solution as a function of total Fe(II) retained from solution in the mineral phase during the 1 mM and 3 mM $Fe(II)_{aq}$ treatments of the batch experiments with USCH (a) and UJAR (b). Data points represent the mean ± standard deviation of duplicate samples.

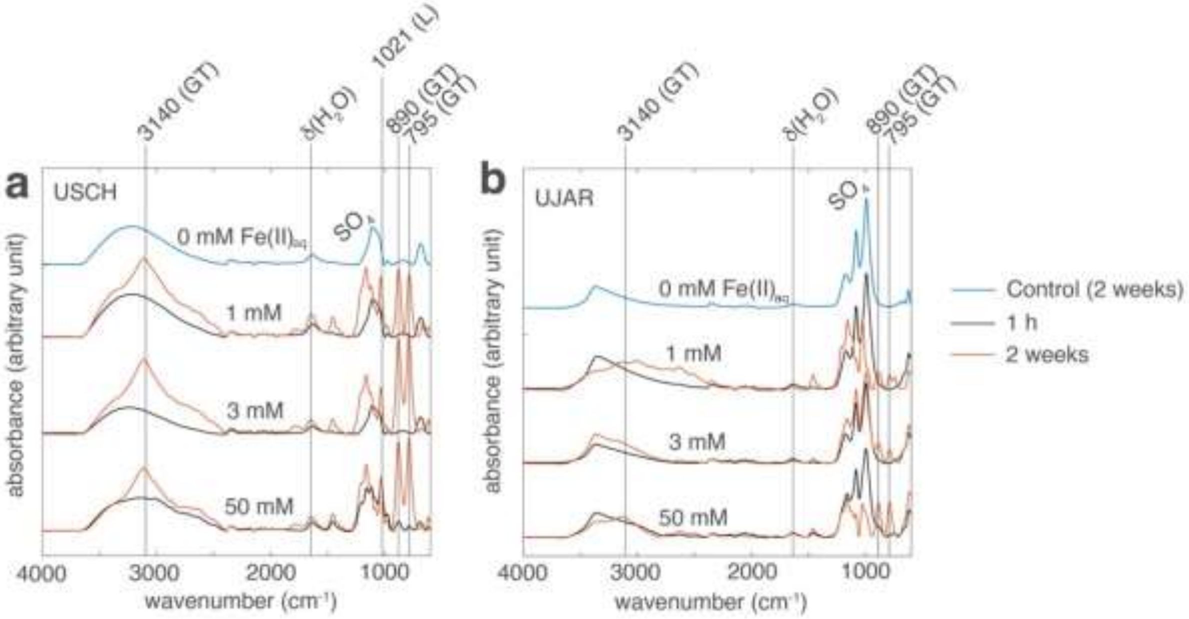

Figure S2. ATR-FTIR spectra of USCH (a) and UJAR (b) reacted with 0 (control), 1, 3, and 50 mM $Fe(II)_{aq}$ for 1 h (black) and 2 weeks (orange), compared with the corresponding 2-week control (blue). Spectra are offset vertically for clarity. Vertical lines mark diagnostic bands for goethite (GT; 3140, 890, and 795 cm^{-1}), lepidocrocite (L; 1021 cm^{-1}), water bending ($\delta(H_2O)$; ~1630 cm^{-1}), and sulfate (SO_4; 1200–1000 cm^{-1} region).

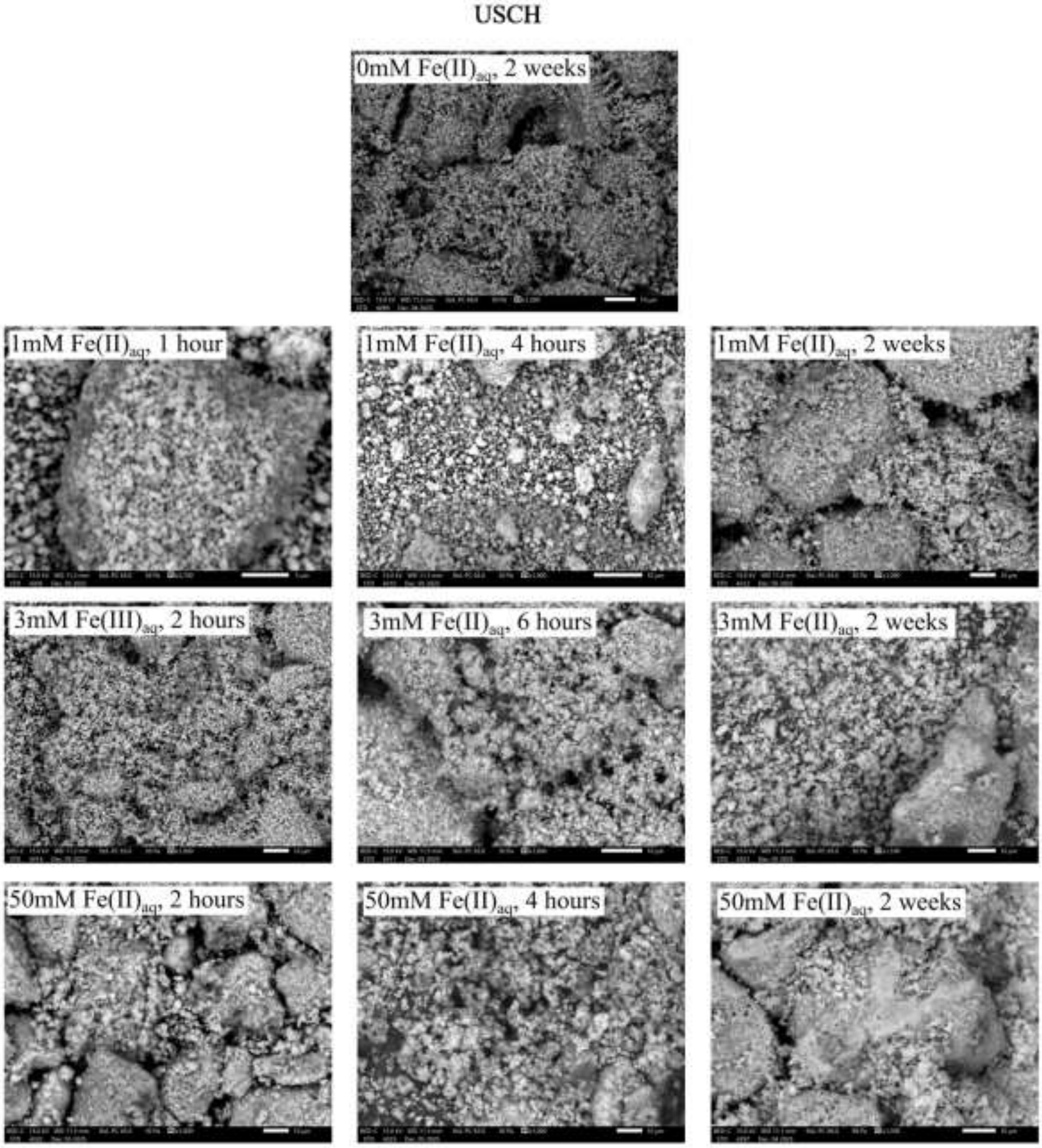

Figure S3. Back-scattered SEM images showing early preservation of near-spherical USCH particles (0-3 mM Fe(II)$_{aq}$, 1-2 h), followed by densification of material and formation of platy aggregates (lepidocrocite) at 4-6 h (1-3 mM Fe(II)$_{aq}$), and progressive growth of goethite within a residual USCH matrix at 2 weeks and 50 mM Fe(II)$_{aq}$.

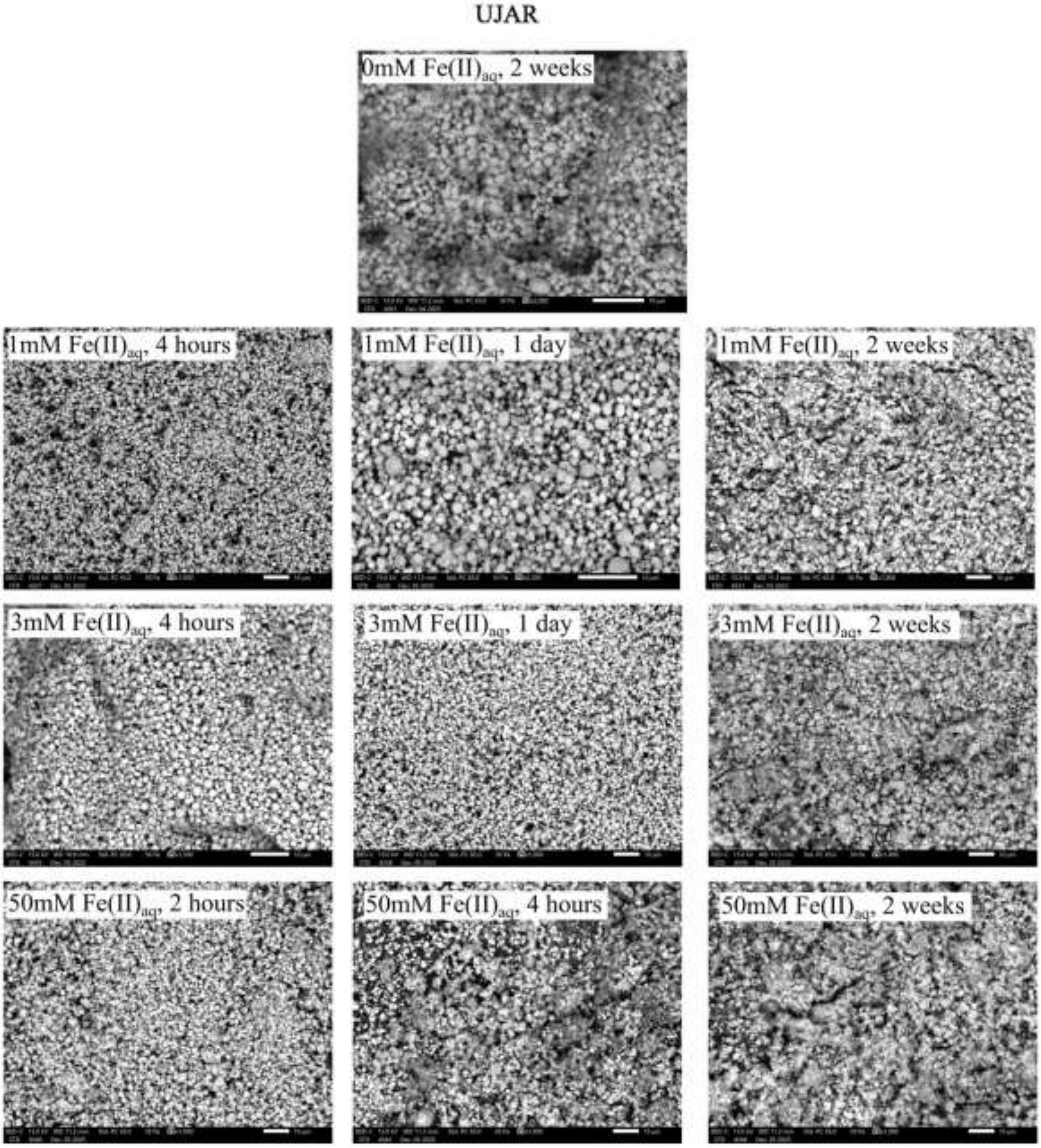

Figure S4. Back-scattered SEM images showing densely packed spheroidal grains of UJAR (0, 1, and 3 mM $Fe(II)_{aq}$, 4 h) and transformation-induced morphological changes, including minor coarsening (1-3 mM $Fe(II)_{aq}$, 1 day; 50 mM $Fe(II)_{aq}$, 2-4 h), and the development of heterogeneous aggregates (lepidocrocite and/or goethite) with increasing Fe(II) loadings and reaction times

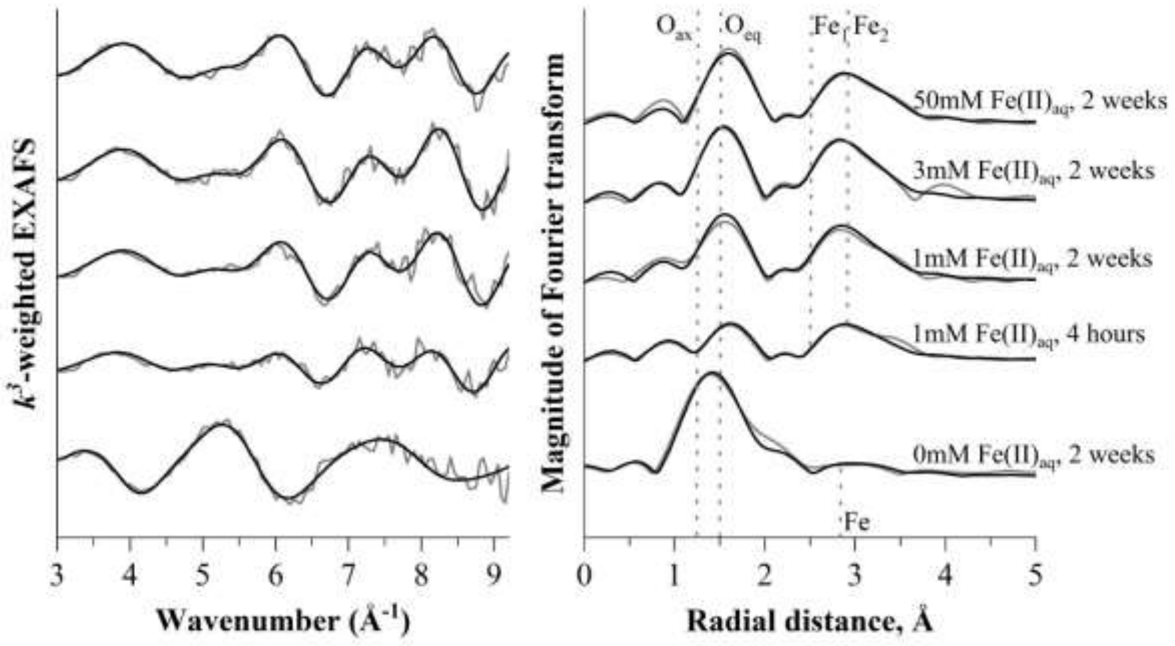

Figure S5. U L_3 edge EXAFS (left) and their Fourier-transform magnitudes (uncorrected for phase shifts, right) for selected USCH samples. The grey lines are the experimental data, while the black lines are the modeled fits.

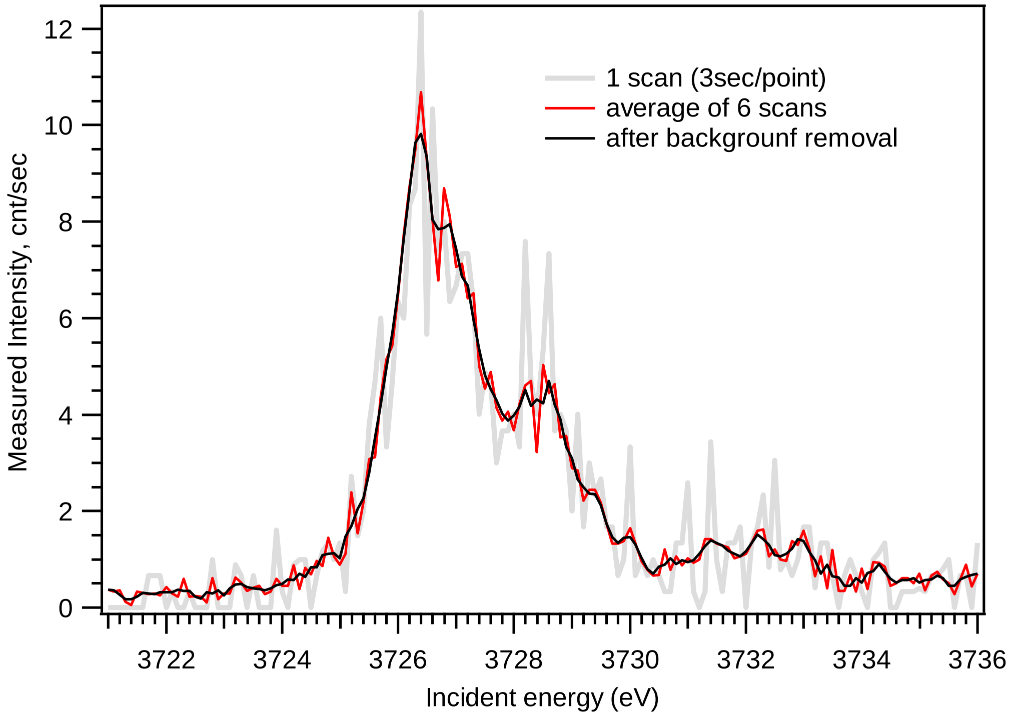

Figure S6. Individual U M_4-edge XANES measurement (single scan), the average of 6 scans and the final spectrum after background subtraction. Sample UJA-50 mM $Fe(II)_{aq}$-4 h.

References

1. Baron, D. & Palmer, C. D. Solubility of jarosite at 4–35 °C. *Geochim. Cosmochim. Acta* **60**, 185–195 (1996).
2. Regenspurg, S., Brand, A. & Peiffer, S. Formation and stability of schwertmannite in acidic mining lakes 1 1Associate editor: C. M. Eggleston. *Geochim. Cosmochim. Acta* **68**, 1185–1197 (2004).
3. Templeton, D. H., Zalkin, A., Ruben, H. & Templeton, L. K. Redetermination and absolute configuration of sodium uranyl acetate. *Acta Crystallogr.* **41**, 1439–1441 (1985).
4. Fernandez-Martinez, A. *et al.* The structure of schwertmannite, a nanocrystalline iron oxyhydroxysulfate. *Am. Mineral.* **95**, 1312–1322 (2010).
5. Boland, D. D., Collins, R. N., Payne, T. E. & Waite, T. D. Effect of Amorphous Fe(III) Oxide Transformation on the Fe(II)-Mediated Reduction of U(VI). *Environ. Sci. Technol.* **45**, 1327–1333 (2011).
6. Scheinost, A. C. *et al.* ROBL-II at ESRF: a synchrotron toolbox for actinide research. *J. Synchrotron Radiat.* **28**, 333–349 (2021).
7. Kvashnina, K. O. & Scheinost, A. C. A Johann-type X-ray emission spectrometer at the Rossendorf beamline. *J. Synchrotron Radiat.* **23**, 836–841 (2016).
8. Solé, V. A., Papillon, E., Cotte, M., Walter, Ph. & Susini, J. A multiplatform code for the analysis of energy-dispersive X-ray fluorescence spectra. *Spectrochim. Acta Part B At. Spectrosc.* **62**, 63–68 (2007).
9. Roßberg, A., Reich, T. & Bernhard, G. Complexation of uranium(VI) with protocatechuic acid application of iterative transformation factor analysis to EXAFS spectroscopy. *Anal. Bioanal. Chem.* **376**, 631–638 (2003).
10. Pan, Z. *et al.* Nanoscale mechanism of UO2 formation through uranium reduction by magnetite. *Nat. Commun.* **11**, 4001 (2020).
